# Diagnostic Problems in Chronic Basophilic Leukemia

**DOI:** 10.4274/tjh.2018.0129

**Published:** 2018-11-13

**Authors:** Cavit Çehreli

**Affiliations:** 1 Dokuz Eylül University Faculty of Medicine, Division of Hematology, İzmir, Turkey

**Keywords:** Myelodysplastic syndrome, Dysplasia, Interleukin-6, Chronic myeloid leukemia, Chronic basophilic leukemia, Mast cell leukemia

## Abstract

Chronic basophilic leukemia (CBL) is an extremely rare type of leukemia. A literature review revealed six cases reported as primary CBL and five patients with secondary CBL. Patients with primary CBL may present with symptoms not related to leukemia. Dysplastic changes in peripheral blood and bone marrow were described and demonstrated in cases of primary and secondary CBL. The literature review also revealed that differential counts made by automated blood cell counters may not characterize cells as basophils in patients with primary and secondary CBL and may mislead physicians in making a differential diagnosis. For these reasons, laboratory studies for the diagnosis of CBL are required, including metachromatic staining by toluidine blue and antigen expressions by flow cytometric analysis, to detect the nature of the neoplastic cells as basophils for a reliable diagnosis of CBL. The literature review failed to reveal specific cytogenetic findings in patients with primary and secondary types of CBL.

## Abstract (tr)

Kronik bazofilik lösemi (KBL) son derece nadir bir lösemi tipidir. Literatürün gözden geçirilmesi ile primer KBL olarak altı olgu ve sekonder KBL olarak beş olgu rapor edildiği görüldü. Primer KBL’li hastalar, lösemi ile ilgili olmayan yakınmalar ile başvurabilirler. Primer ve sekonder KBL hastalarının periferal kan ve kemik iliği yaymalarında anormal değişiklikler tanımlandı ve gösterildi. Literatür taraması, primer ve sekonder KBL hastalarında otomatik kan hücre sayıcıları tarafından yapılan ayırıcı sayımların hücreleri bazofil olarak karakterize edemeyeceğini ve doktorları ayırıcı tanıda yanıltabileceğini ortaya koymuştur. Bu nedenle güvenilir bir KBL tanısı için neoplastik hücre türünün bazofil olarak saptanması, toluidine mavisi ile metakromatik boyanmayı ve membranlarındaki antijen ekspresyonlarını akım sitometrik analizi ile yapan kanıtlayıcı laboratuvar incelemelerini gerektirir. Literatürün gözden geçirilmesi primer ve sekonder KBL hastalarında özel sitogenetik bulgular olmadığını göstermiştir.

## Introduction

Basophils are one of the members of granulocytes in the myeloid lineage and are formed by proliferation and differentiation of committed myeloid progenitors. Basophils in peripheral blood (PB) or tissues range in size from 10 to 15 µm and have nuclei that are purplish or dark blue and cytoplasmic granules of dark blue to purple and even blackish color as seen on Wright-stained PB and bone marrow (BM) smears [1]. A review of the international literature revealed 6 cases reported as primary chronic basophilic leukemia (CBL) [2,3,4] and 5 cases reported as chronic myeloid leukemia (CML) with transformation to CBL, as a secondary CBL [5,6,7,8,9]. The purpose of this review is to remind readers of the importance of diagnostic problems as automated blood cell counters (ABCCs) may not characterize cells as basophils in patients with primary and secondary CBL, but may simply flag them [3,4,9]. The literature review failed to reveal lymphoma or non-hematologic neoplasia with transformation to secondary CBL.

Age and sex distribution, splenic size, presenting symptoms, results of complete blood counts, and cytogenetic and molecular studies in patients with primary and secondary CBL are shown in Table 1 and Table 2, respectively.

### Presenting Symptoms

Presenting symptoms may not be related to leukemia, as in Case #6 with primary CBL (Table 1). The patient presented with recurrent occurrence of febrile episodes and abdominal pain at about 8-week intervals, associated with simultaneous cyclic oscillation in neutrophil leukocyte counts and in the levels of C-reactive protein (CRP) when leukocyte counts climbed to the peak level and remained with the consumption of analgesics-antipyretics in 2 h. Physicians were misled in making a differential diagnosis of familial Mediterranean fever because they relied on differential counts made by ABCCs [4]. However, manual differential counts made on PB smears during a febrile episode revealed that the ABCCs wrongly characterized 70% of basophils as neutrophils; this was confirmed by toluidine blue stain and antigen expression by flow cytometric analysis. Marked elevation in plasma interleukin-6 (IL-6) level of 15.8 pg/mL (normal: <5.8 pg/mL) was detected during the febrile episode. Real-time polymerase chain reaction showed IL-6 gene expression in neoplastic basophils, revealing that IL-6 was released from neoplastic basophils infiltrating the BM [4] (Table 1). IL-6 production and release by normal human basophils has not been reported in the literature [10].

### Values of PB and BM Basophil Percentages in the Diagnosis of CBL

As shown in Tables 1 and 2, BM basophil percentages of 15%, 26%, and 20% in 3 of 4 patients with primary CBL were reported by Pardanani et al. [2]. These values are almost equal to the BM basophil percentage of ≥20% in 11% of 25 patients in the accelerated phase of CML reported by Kantarjian et al. [11] and also BM basophil percents greater than 1% (range 1 to 27%) in 34 cases and median 19% (range 14 to 27%) in 6 patients (in 4 of whom toluidine blue staining was positive) were reported by Hoyle et al. [12] in their series of 750 patients with acute myeloid leukemia. However, the BM basophil percentages in patients reported by Pardanani et al. [2] were markedly lower compared to the BM basophil percentages (40%,75%) of two patients in the chronic phase [3,4] and the value recorded (51%) in a patient in the accelerated phase of primary CBL [5] (Table 1). BM basophil percentages of 55%, 51.4%, 63%, 72%, and 66% in six patients with secondary CBL were also respectively recorded (Table 2) [6,7,8,9].

### Dysplastic Changes in Primary CBL

In Case #5, a patient with primary CBL, dysplasia in the PB smear included hypogranular basophils with small and fine granulations and nuclear hyperlobation. In the BM smear, an increase in cellularity and megakaryocytes and hypogranular, agranular, and hypersegmented forms of basophils and eosinophils with coarse granulations and nuclear hyperlobation in addition to megakaryocytes with many small and hypolobated forms were reported [3].

In Case #6, a patient with primary CBL in the chronic phase, dysplasia appeared as basophils with coarse basophilic granules, occasionally hypersegmented or giant segmented, and band forms of basophils in PB smear [4].

**Figure 1:**
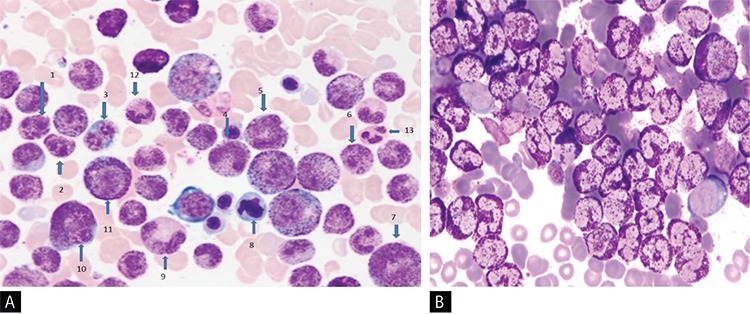
A) Showing hyposegmented basophils (1, 2, 6), binuclear erythroblast (3), giant forms of basophilic bands (4, 5), large eosinophilic myelocyte (7), erythroblast with dysplastic nucleus (8), giant basophilic hypogranular metamyelocyte (9), giant binuclear basophilic metamyelocyte (10), basophilic myelocyte (11), neutrophilic band (12), segmented neutrophil (13) in chronic phase of primary chronic basophilic leukemia (Wright’s stain, 100^x^); B) Demonstrating red color (metachromatic) granular cytoplasmic staining in 70% nucleated cells of the bone marrow (toluidine blue stain, 100^x^).

In the BM smear, an increase in cellularity, megakaryocytes, eosinophils, and prominent basophilic hyperplasia with the presence of all stages of maturation that resulted in marked neutrophilic suppression and mild suppression in erythroid lineages were noted. Three-lineage dysplasia manifested as hyposegmented basophils, giant segmented bands, and metamyelocyte forms of basophils and hypogranular basophilic metamyelocytes were noted. Dysplasia seen in eosinophils included binuclear metamyelocyte and myelocyte forms of eosinophils and large eosinophilic myelocytes. Occasional binuclear (Figure 1A) and multinuclear red cell precursors were also noticed. In addition, binuclear agranular immature megakaryocytes, mononuclear giant forms of megakaryocytes, and megakaryocytes with nuclear hyperlobation were observed. Toluidine blue stain showed red (metachromatic) granular staining in about 75% of non-erythroid granular cells in the marrow fields (Figure 1B).

### Dysplastic Changes in the Accelerated Phase of Primary CBL

**Figure 2:**
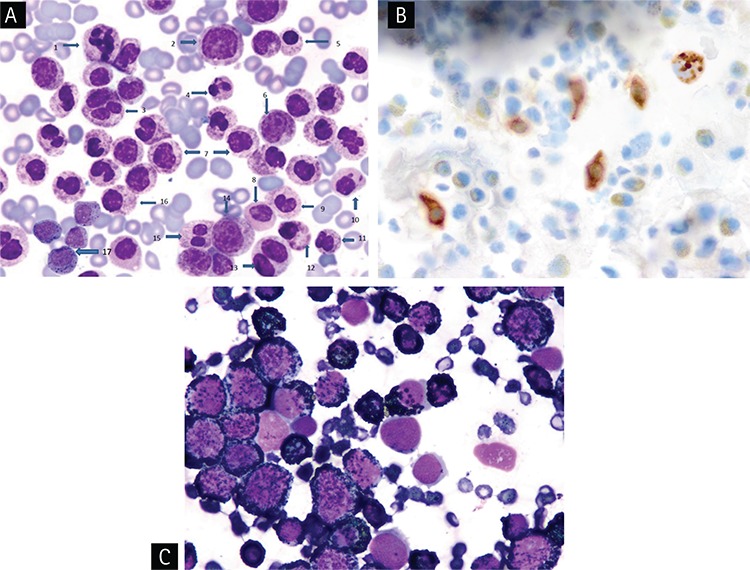
A) Demonstrating hypersegmented basophil (1), basophilic myelocyte (2), giant binuclear basophilic metamyelocyte (3), pyknotic eosinophil and basophil with drum-stick like nuclear sticks (4, 10), normal basophilic metamyelocyte (6), pyknotic myelocyte, metamyelocytes, binuclear basophilic metamyelocyte and basophilic myelocyte (5, 7, 11, 12, 16), agranular and hypogranular metamyelocyte (8, 9), binuclear hypogranular metamyelocyte (13), basophilic myelocyte (14), Pelger-Hüet anomaly (15) and aggregates of mast cells having mixed orange and dark purplish to black color round cytoplasmic granules (17) in accelerated phase of primary chronic basophilic leukemia with mast cell leukemia (Wright’s stain, 100^x^); B) Showing tryptase activity in the round, brown color of cytoplasmic granules of mast cells demonstrated by immunohistochemical staining for tryptase. (tryptase immunohistochemical staining, 100^x^); C) Demonstrating black granular cytoplasmic staining by peroxidase stain in myeloperoxidase-positive basophils and absence of staining in aggregates of cells representing myeloperoxidase-negative mast cells in the bone marrow (peroxidase stain, 100^x^).

Clinical and hematologic features of the accelerated phase of primary CBL were only observed by Cehreli et al. [5] in their patient in the chronic phase of primary CBL (Case #6) after 53 months of hematologic remission; this is shown as Case #7 in Table 1. She presented with symptoms of anemia and was found to have relapse of her CBL and development of systemic mastocytosis (SM) as a secondary neoplasia. Three months later, the patient showed a rapid downhill clinical course when transformation of primary CBL to the accelerated phase with simultaneous occurrence of mast cell leukemia (MCL) was detected [5]. She experienced febrile episodes with abdominal pain during the accelerated phase of primary CBL with MCL. Although mast cells and eosinophils were shown to produce IL-6 [10], no febrile episodes were observed despite increases in mast cell (MC) counts to 3x10^9^/L and eosinophil counts to 5.3x10^9^/L unless her basophil counts climbed to >40x10^9^/L when prominent elevation in IL-6 level (38.5 pg/mL) was found. In a Wright-stained BM smear, basophilic hyperplasia with the presence of all stages of maturation that resulted in marked suppression in neutrophilic and erythroid lineages in addition to aggregates of MCs with a new and undefined MC morphology were demonstrated. MCs have round or oval nuclei, one or more nucleoli in immature forms, and mixed orange and dark purplish to black round cytoplasmic granules (Figure 2A). Tryptase immunohistochemical staining of the PB smear showed round, brown, granular cytoplasmic staining in the aggregates of the cells, confirming that these cells demonstrated tryptase activity and represented MCs (Figure 2B), because b-tryptase is a natural serine protease and is the most abundant mediator stored in the granules of MCs [13]. Three-lineage dysplasia manifested as giant hypersegmented basophils, giant binuclear metamyelocytes, binuclear hypogranular basophilic metamyelocytes, binuclear erythroblasts, and Pelger-Hüet anomalies were noted. Additionally, marked pyknosis, manifested as a decrease in both cellular and nuclear sizes, resulted in dense chromatin clumping, inducing a nuclear appearance that resembled a chromatin mass. Pyknotic myelocytes, metamyelocytes, binuclear basophilic metamyelocytes, and drum stick-like nuclear sticks in both pyknotic eosinophils and basophils were observed (Figure 2A). Dysplastic changes in the megakaryocytic lineage were similar to those seen in the chronic phase of the patient [4].

Marked pyknotic changes in the accelerated phase of primary CBL [5] have not been reported in patients with the chronic phase of primary CBL [3,4] as well as in patients with the accelerated [5,7,8] and chronic phase [9] of secondary CBL in the literature.

### Dysplastic Changes in the Chronic and Accelerated Phases of Secondary CBL

Case #5 in Table 2 was the only patient in the chronic phase of secondary CBL presenting with dysplasia. Diagnosis of CML may possibly be made when transformation of CML to CBL occurs; in this case, the patient had marked basophilia with the presence of 40% and 66% basophils in PB and BM, respectively, and antigen expressions by flow cytometric analysis revealed the nature of cells as basophils. Dysplasia included cytoplasmic hypogranulation or agranulation and nuclear hypersegmentation, eosinophils with abnormal granulation and nuclear hyperlobation, and dyserythropoiesis [9]. No dysplastic findings have been reported in patients in the accelerated phase of secondary CBL [6,7,8] (Table 2).

### Confirmatory Laboratory Studies

Both basophils and MCs have electron-dense cytoplasmic granules and produce numerous mediators such as histamine common to both cells. They also both show metachromatic staining with basic dyes, toluidine blue, and Alcian blue [1]. Toluidine blue stain showed a red (metachromatic) granular cytoplasmic staining in both basophils and MCs (Figure 1B) [4,5,14,15]. Peroxidase stain showed black granular cytoplasmic staining in basophils, but MCs do not contain myeloperoxidase and showed negative activity by peroxidase stain (Figure 2C) [16]. Flow cytometric analysis of mononuclear cells (MNCs) of the BM using monoclonal antibodies against the following antigens in Cases #6 and #7 showed that antigen expressions were positive for CD10 (dim), CD11c (dim), CD13, CD15, CD22 (dim), CD25, CD33, CD38, CD45, CD123, immunoglobulin D (IgD) receptor, and myeloperoxidase and negative for HLA-DR, CD7, CD34, CD71, and CD117 with aberrant expression of CD10 [17,18], thus revealing that the neoplastic cells were basophils.

Expression of the IgD receptor on normal basophils was demonstrated by Chen and Cerutti [19] and was also shown on neoplastic basophils by Cehreli et al. [4,5].

### Importance of Confirmatory Laboratory Studies for the Diagnosis of CBL

Tang et al. [3] and Vaidya et al. [9] reported that automated hematology analyzers did not characterize cells as basophils, but simply flagged them. Flow cytometric immunophenotyping became particularly important in confirming the nature of cells as basophils [9]. Cehreli et al. [4,5] reported that ABCCs, even with advanced technology, wrongly characterized basophils as neutrophils, misleading physicians in making differential diagnosis when the physicians relied on differential counts made by ABCCs. These reported observations suggest that manual differential counts should be seen before making a decision for diagnosis and also confirmed by metachromatic staining with toluidine blue stain in PB or BM smears and antigen expressions by flow cytometric analysis in BM MNCs to make an accurate diagnosis of CBL. The authors also proposed that neoplastic basophils with coarse, dark purple basophilic granules as demonstrated in Figure 1A may possibly mimic neutrophils with toxic granules [5]. Neutrophils usually contain light purplish-blue fine granules on Wright-stained PB smears (Figure 1A). Interestingly, in 1932, Kugel and Rosenthal [20] found that during bacterial infections fine neutrophilic granules are replaced by large, dark, irregular basophilic granules, which are called toxic granules, compared to the fine granules of the neutrophils.

### Oscillation in Leukocyte Counts

Similar to cyclic oscillations in leukocyte (neutrophil) counts reported in patients with CML [21,22], cyclic oscillations in basophil counts with simultaneous elevations in CRP levels and association with febrile episodes were only demonstrated by Cehreli et al. [5] in patients with primary CBL [4], but not reported in patients with primary [3] and secondary CBL [6,7,8,9].

Literature findings reveal that the diagnosis of CBL is mainly based on basophil morphology and increase in PB and BM basophil percentages. The literature also suggests that higher BM and PB basophil percentages are required to establish a satisfactory morphologic diagnosis of CBL. Based on the reported literature findings [2,3,4,5,6,7,8,9], diagnostic criteria for CBL as shown in Table 3 can be proposed.

### Differential Diagnosis

The presence of an increase in megakaryocytes with atypical megakaryocytic hyperplasia and BM basophil percentages of less than 40% in 3 of four patients (Cases #1-3), the absence of diagnostic confirmatory laboratory studies (Table 1), and an abnormal pattern of perivascular atypical MC infiltration detected by tryptase immunohistochemical staining (Cases #2 and #4) suggesting concurrent MC disease were reported as primary CBL by Pardanani et al. [2]. These findings created diagnostic problems in classifying the cases as primary CBL as described by Pardanani et al. [2] because according to the proposed diagnostic criteria for CBL (Table 3) BM basophil percentages were less than ≥40%, increased megakaryocytes with dysplasia have been described in patients with essential thrombocythemia and chronic idiopathic myelofibrosis [23], and presence of abnormal pattern of perivascular atypical MC infiltration detected by tryptase immunohistochemical staining in the BM biopsy is one of the diagnostic criteria for SM [24].

Dysplastic changes described in two patients in the chronic phase of primary CBL [3,4] and in a patient in the accelerated phase of primary CBL [5] (Table 1) in addition to a patient in the chronic phase of secondary CBL [9] (Table 2) were not specific for CBL and have been described in patients with myelodysplastic syndrome (MDS) [25,26]. In MDS, dysplastic changes are accompanied by specific cytogenetic abnormalities [25], whereas cytogenetic studies specific for primary and secondary CBL have not been reported in the literature [2,3,4,5,6,7,8,9]. Additionally, laboratory studies with metachromatic staining by toluidine blue stain in both primary and secondary CBL [4,5,7,8] and antigen expressions detected by flow cytometric analysis in both primary and secondary CBL [3,4,5,9] are confirmatory for diagnosis of CBL (Tables 1 and 2), but have no diagnostic value in patients with MDS [25,26]. Patients in the chronic and accelerated phases of CML associated with both PB and BM basophil percentages of ≥40% [6,7,8,9], patients with autoinflammatory diseases manifesting with recurrent attacks of fever and abdominal pain [27] and additionaly, chronic myeloproliferative disorders [28], mastocytosis variants [29], Castleman’s disease [30] and the possibility of underlying CBL [4,5] should be considered in the differential diagnosis in the patients presented with progressive leukocytosis associated with eosinophilia and elevations in IL-6 and CRP levels.

## Conclusion

ABCCs may not characterize cells as basophils in patients with primary and secondary CBL [3,4,5,9] and may mislead physicians in making a differential diagnosis [4]. The new generation of blood cell counters could be designed to contain toluidine blue stain for the detection of neoplastic basophils, and MCs with atypical or new MC morphology will be beneficial in leading physicians to make a reliable differential diagnosis, like ABCCs containing methylene blue stain for reticulocyte counts. Diagnosis, treatment, and follow-up should be performed with the guidance of manual differential counts in cases of primary and secondary CBL. The frequency and type of dysplastic changes [3,4] observed, especially during the accelerated phase of primary CBL [5] (Figures 1 and 2), are comparable to those seen in patients with MDS and may create problems in the differential diagnosis of MDS [25,26].

## Figures and Tables

**Table 1:**
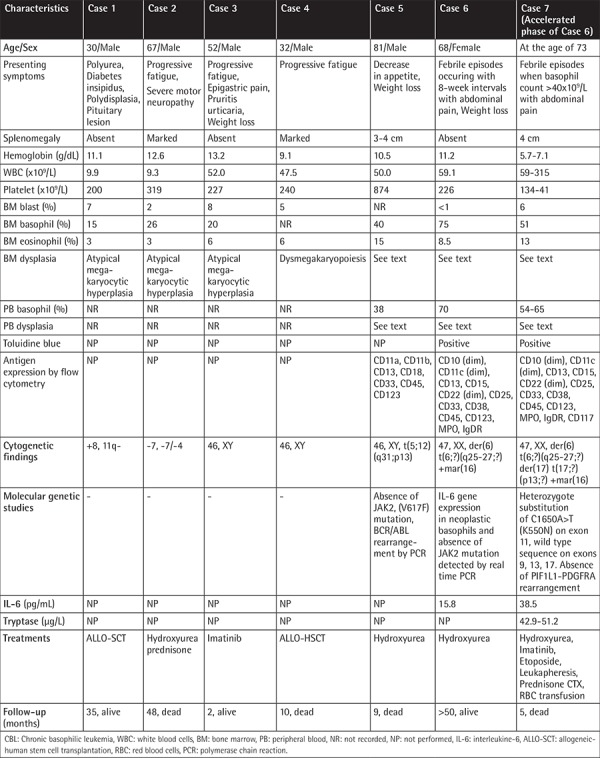
Characteristics of chronic and accelerated phases of primary chronic basophilic leukemia patients [2,3,4,5].

**Table 2:**
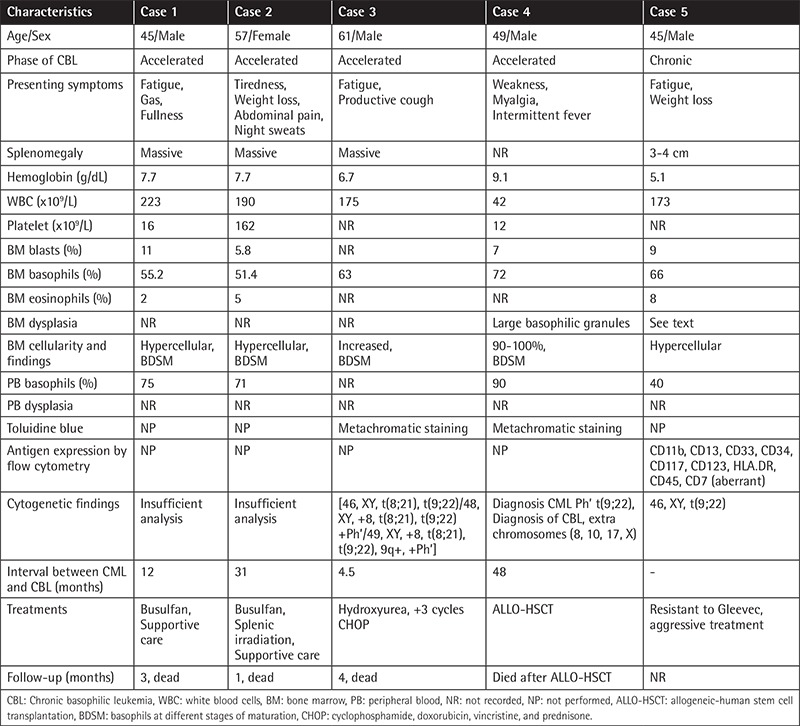
Characteristics of secondary chronic basophilic leukemia patients reported in the literature [6,7,8,9].

**Table 3:**
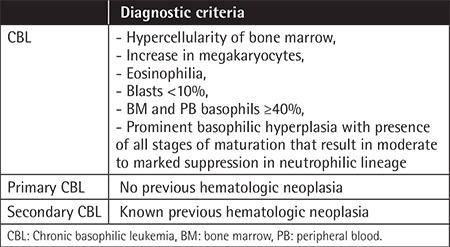
Proposed diagnostic criteria for chronic basophilic leukemia.
